# Precision oncology meets Generative AI: assessing large language models in multidisciplinary GIST tumor boards

**DOI:** 10.1186/s12885-026-16537-7

**Published:** 2026-08-01

**Authors:** Reza Dehdab, Judith Herrmann, Fiona Mankertz, Patrick Ghibes, Nour Maalouf, Sebastian Werner, Jens Strohäker, Katrin Benzler, Lars Zender, Saif Afat, Konstantin Nikolaou, Christoph K. W. Deinzer

**Affiliations:** 1 Department of Radiology, University Hospital Tübingen, University of Tübingen, Tübingen, Germany; 2 Department of General, Visceral- and Transplant Surgery, University Hospital Tübingen, Tübingen, Germany; 3 Department of Internal Medicine VIII - Medical Oncology and Pneumology, University Hospital Tübingen, Tübingen, Germany; 4 Center for Soft Tissue Sarcomas, GIST and Bone Tumors (ZWS) of the University Hospital Tübingen and the Comprehensive Cancer Center (CCC) Tübingen-Stuttgart, Tübingen, Germany

**Keywords:** Gastrointestinal stromal tumor (GIST), Artificial intelligence (AI), Large language models (LLMs), Multidisciplinary tumor boards (MTB), Clinical decision support

## Abstract

**Objectives:**

Gastrointestinal stromal tumors (GISTs) are molecularly heterogeneous neoplasms whose management depends on individualized, multidisciplinary decision-making. While multidisciplinary tumor boards (MTBs) represent the standard of care, access remains limited in many clinical settings. This study evaluates the performance of two large language models in generating GIST MTB recommendations and assesses their agreement with expert MTB decisions using predefined clinical evaluation criteria.

**Materials and methods:**

This retrospective single-center study included 99 GIST cases discussed at an institutional MTB. A structured prompt was developed to extract clinical variables and generate treatment recommendations. ChatGPT-5 and Qwen3 were independently evaluated across five predefined domains: diagnostic recommendations, therapeutic modalities, treatment sequence and timing, systemic therapy regimen selection, and clinical contextualization. Two expert reviewers scored all outputs in a blinded fashion. Normalized scores, inter-model comparisons, perfect-case rates, and inter-rater agreement were analyzed.

**Results:**

Both models demonstrated high concordance with expert MTB recommendations, with mean total normalized scores of 0.901 for ChatGPT-5 and 0.875 for Qwen3, without a significant difference between models (*p >* 0.05). Perfect agreement was observed in 52.5% of ChatGPT-5 cases and 48.5% of Qwen3 cases (*p >* 0.05). Diagnostic recommendations scored significantly lower than all other domains in both models (all adjusted *p <* 0.05). Overall inter-rater agreement was almost perfect (weighted Cohen’s kappa=0.978).

**Conclusions:**

Both models demonstrated high agreement with expert GIST MTB recommendations, with no significant performance difference between them. Diagnostic reasoning represented the weakest domain, reflecting the challenge of reconstructing context-dependent workup decisions from tumor board documentation. These findings support a potential assistive role for LLMs in GIST MTB workflows, while underscoring the continued necessity of expert oversight.

## Background

Gastrointestinal stromal tumors (GISTs), which arise from mesenchymal precursor cells, represent the most common subtype of soft tissue sarcomas of the gastrointestinal tract and account for approximately 1–2% of all primary gastrointestinal malignancies [1]. GISTs arise most commonly in the stomach (60–70%), followed by the small intestine (20–30%), while colorectal (5%) and esophageal (< 5%) locations are less frequent [2]. Clinically, they are characterized by a wide spectrum of malignant potential, ranging from virtually indolent tumors to highly aggressive cancers [3]. Because of the variable malignant potential and the mutation-dependent response to targeted therapy, clinical decision-making in GIST requires an individualized, risk-adapted approach [4].

Given the molecular heterogeneity and the broad spectrum of clinical behavior observed in GISTs from indolent, low-risk tumors to aggressive, high-risk metastatic disease, management typically requires the input of multiple specialties [5]. Accordingly, GIST management nearly always benefits from discussion in multidisciplinary tumor boards (MTBs), particularly in complex or borderline cases [1]. Given the rarity of sarcoma entities and the need for specialized expertise, diagnosis and treatment are recommended to be performed in certified sarcoma centers or in close collaboration with such centers [6–9]. However, implementation of MTBs is not without limitations. In many settings, particularly in low-resource regions or smaller institutions, access to expert MTBs may be limited due to logistical constraints, time demands, lack of subspecialized expertise, or geographic disparities [7, 10]. Even in high-income countries, increasing case complexity and growing diagnostic data volumes have placed significant strain on MTB workflows [11]. These challenges have prompted interest in AI-based decision support systems that may complement MTB workflows and support multidisciplinary decision-making [12].

Recent advances in this field have enabled large language models (LLMs) to interpret clinical narratives, synthesize information across medical domains, and generate context-aware recommendations [13]. Models such as ChatGPT (OpenAI) have demonstrated the ability to simulate expert-level reasoning and produce structured outputs from unstructured inputs, including radiology findings, pathology reports, and clinical histories [14]. These capabilities have opened up new possibilities for integrating LLMs into multidisciplinary workflows, particularly in oncology, where clinical complexity and data volume continue to increase [15].

Despite growing enthusiasm, the application of LLMs in tumor board settings has been sparsely studied and remains largely exploratory. Early studies have shown variable performance, with concerns about reliability, hallucinations, and lack of clinical nuance [16–18]. Although LLMs have recently been evaluated in GIST-related tumor board settings, prior work has not assessed their performance using predefined, clinically relevant evaluation criteria that separate the different components of MTB decision-making. Therefore, systematic evidence on how LLM-generated recommendations align with expert MTB decisions in GIST remains limited.

The objective of this study was to assess the performance of LLMs in the context of real-world GIST MTB cases, using predefined, clinically relevant evaluation criteria. By analyzing the degree of alignment between model-generated outputs and expert consensus in GIST management, this study evaluates LLM performance across individual components of MTB decision-making. The findings may help define the current capabilities and limitations of such models in complex clinical workflows and inform future integration strategies within AI-assisted MTB environments.

## Materials and methods

### Study design and ethical approval

This retrospective observational single-center study was conducted at the University Hospital Tübingen and the Comprehensive Cancer Center Tübingen-Stuttgart, Tübingen. Ethical approval was obtained from the Institutional Review Board of the University of Tübingen (Ethics Committee of the Faculty of Medicine of the University of Tübingen, reference no. 418/2024BO2). Clinical trial registration was not applicable. Due to the retrospective design and the use of fully anonymized clinical data, the requirement for informed consent was waived. All procedures were performed in accordance with the Declaration of Helsinki. The study was reported in accordance with the Checklist for Artificial Intelligence in Medical Imaging (CLAIM) where applicable.

### Patient selection

**Fig. 1:**
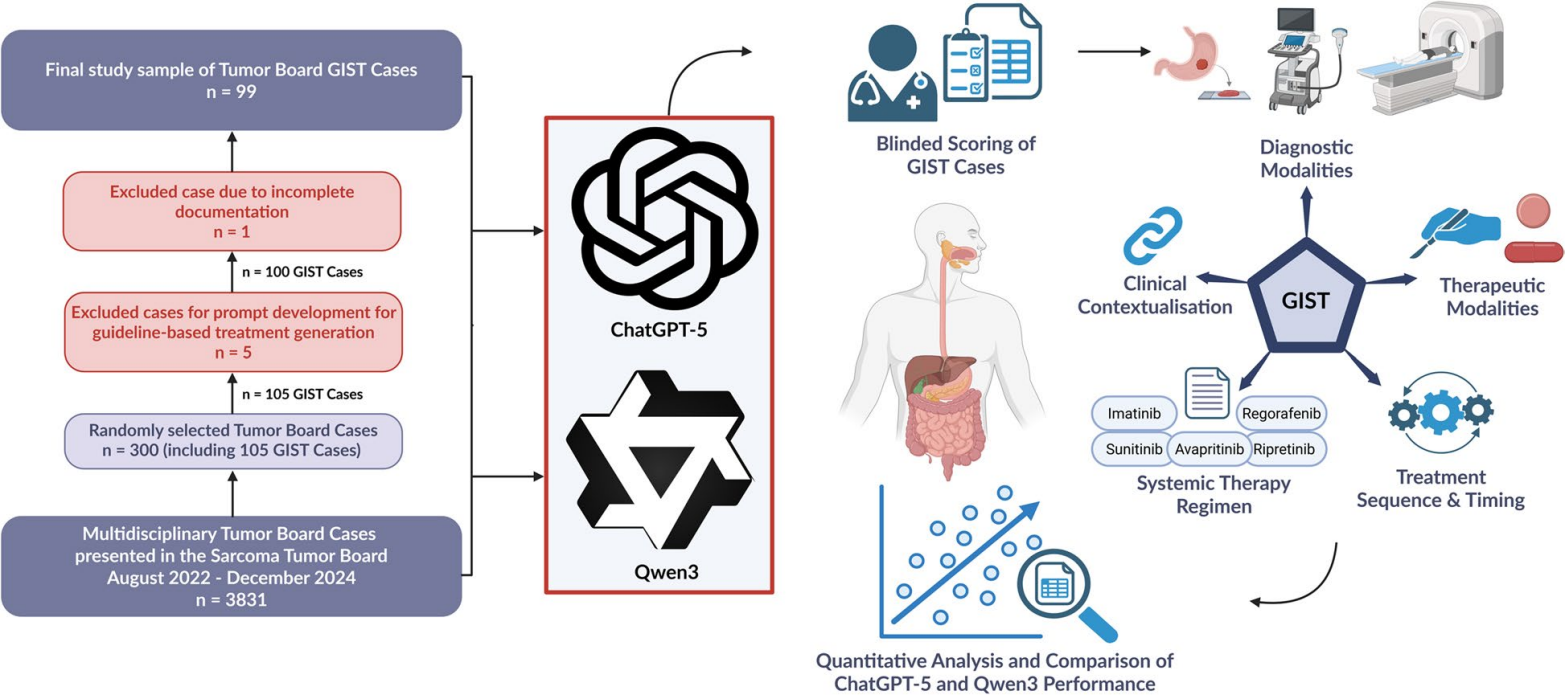
Schematic representation of the study workflow

All cases were discussed in the multidisciplinary tumor board of the certified high-volume sarcoma center at the University Hospital Tübingen and the Comprehensive Cancer Center Tübingen-Stuttgart between August 2022 and December 2024 and were retrospectively retrieved from the institutional sarcoma tumor board registry. Subsequently, 300 cases were selected using a computer-generated random number sequence. This sample size was chosen to provide a feasible real-world cohort for detailed manual case extraction, dual expert scoring, and paired model comparison across five predefined evaluation criteria. GIST cases within this randomly selected cohort were then identified and assessed for eligibility. Eligible cases required a documented tumor board recommendation and sufficient clinical information to allow standardized case extraction and subsequent evaluation of model-generated recommendations. Cases were considered ineligible if the tumor board recommendation was unavailable or if key information required for case reconstruction, including diagnosis, molecular findings, imaging results, treatment history, or clinical status, was missing from the referral documentation. Eligibility and case completeness were assessed by two study investigators with sarcoma expertise ([C.K.W.D.], [K.B.]) prior to model evaluation. An overview of the study workflow is illustrated in Fig. 1 [19].

### Prompting

For the GIST-specific evaluation, a dedicated prompt was developed to support standardized extraction of case information and generation of guideline-concordant treatment recommendations. The prompt was prepared by two authors ([R.D.] and [C.K.W.D.]) and finalized through consensus discussion, based on current SEOM-GEIS and ESMO guidance for the management of GISTs [6, 8].

Before application to the final study cohort, the prompt was piloted on five GIST cases retrieved from the institutional multidisciplinary tumor board archive. These cases were used only for prompt development and were not included in the final analysis. All pilot cases contained complete tumor board decisions, which allowed the authors to assess whether the prompt captured clinically relevant information and produced recommendations consistent with established GIST management pathways. Based on review of the generated outputs, the prompt was refined to improve the completeness and structure of clinical data extraction and to clarify the decision-making instructions. In particular, the extraction section was revised to explicitly request clinically relevant variables required for GIST decision-making, including disease extent, resection status, mutation profile, predicted imatinib sensitivity, prior systemic treatment, treatment response, and patient-specific clinical factors. The recommendation section was refined to require explicit differentiation between localized and metastatic disease, resectable and unresectable tumors, and first-line versus subsequent-line systemic treatment settings. Final tumor board decisions from the pilot cases were used solely to verify that the prompt captured the information required for guideline-based decision-making and were not used to introduce case-specific instructions. Following these refinements, the prompt was finalized and remained unchanged throughout evaluation of the study cohort. The pilot cases were deliberately selected to reflect the clinical heterogeneity of GIST and varied with regard to primary tumor site, tumor size, mitotic activity, mutational profile, disease extent, resectability, previous treatment exposure, and treatment response. This development step was intended to ensure that the final prompt was applicable to both localized and advanced disease scenarios encountered in routine GIST tumor boards.

The final prompt followed a two-part structure. First, the LLM was instructed to extract predefined clinical variables from the free-text tumor board registration letters. These variables comprised diagnosis and disease extent, primary tumor location, tumor size, mitotic rate, risk category, resection status, molecular findings, predicted imatinib sensitivity, imaging findings relevant to resectability or progression, previous treatments, treatment response, and patient-specific clinical factors such as comorbidities, contraindications, or treatment tolerability.

Second, the LLM was instructed to formulate a treatment recommendation according to guideline-based GIST management. The recommendation was required to account for disease status, mutational profile, sensitivity or resistance to imatinib, surgical resectability, and prior systemic treatment. The prompt specifically required the model to distinguish localized from metastatic disease, identify indications for surgery, neoadjuvant imatinib, adjuvant imatinib, mutation-directed therapy, and subsequent-line tyrosine kinase inhibitor treatment in the setting of progression. When appropriate, the model was also instructed to consider clinical trial enrollment or best supportive care.

**Fig. 2:**
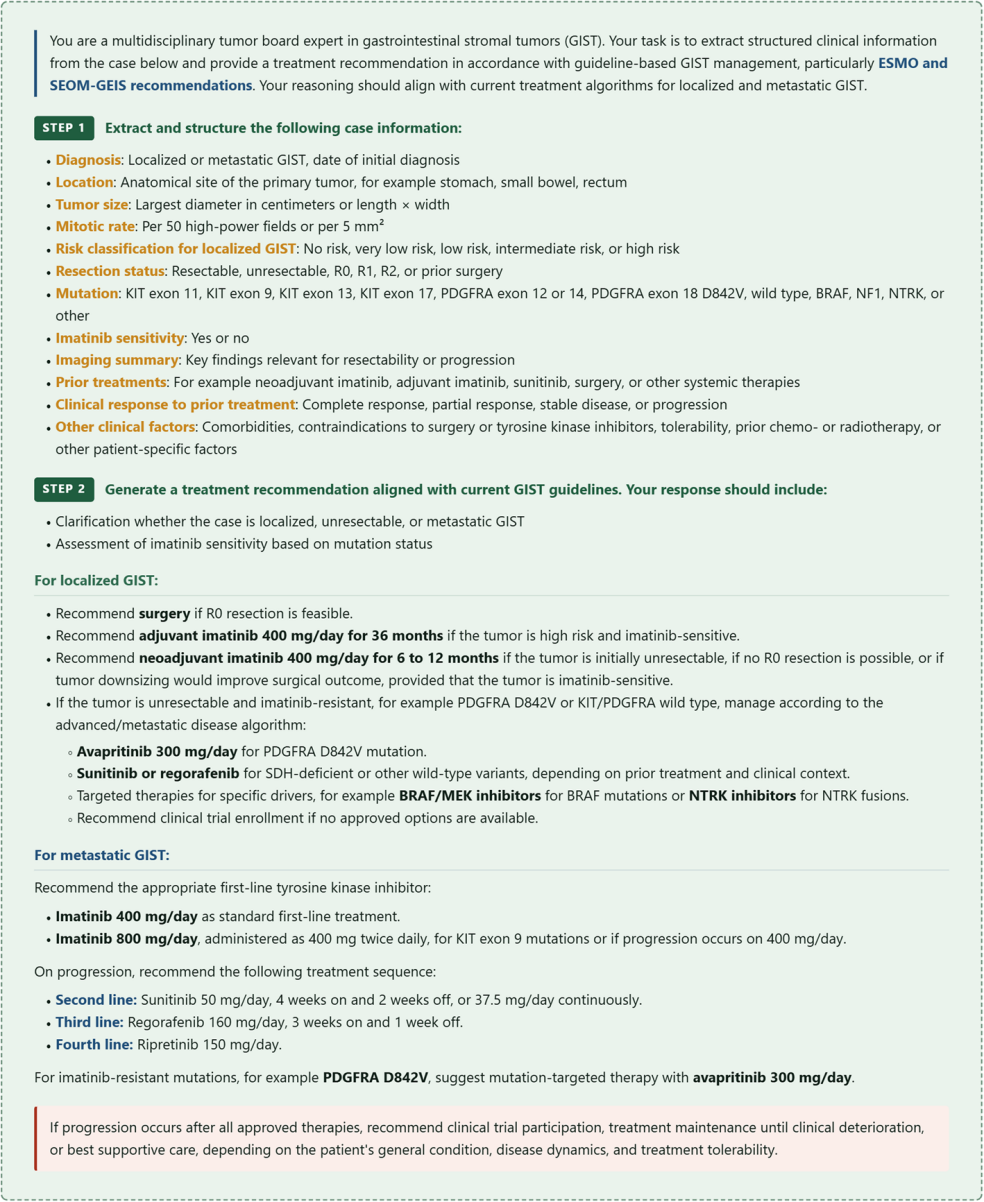
Structured text prompt developed for LLM-based generation of guideline-concordant GIST tumor board recommendations

The final prompt was applied to the selected LLM through the official web interface without model fine-tuning, additional software, or external retrieval tools. ChatGPT-5 (OpenAI) [20] and Qwen3 (Alibaba Cloud) [21] were accessed during the period from October to December 2025. Each case was evaluated in a new, independent chat session to avoid carry-over effects from previous interactions. Browser-based search, model memory functions, and retrieval augmentation were not used during the evaluation. All evaluations were performed using the default interface settings of the respective platforms, including the default temperature and inference settings. Because the original tumor board documentation was written in German, all case inputs were entered in German. Each case was submitted independently in a single uninterrupted interaction, and no manual correction, feedback, or prompt modification was performed during the evaluation process. An example tumor board case and the corresponding model output are provided in Fig. 2.

### Evaluation criteria and point allocation

**Fig. 3:**
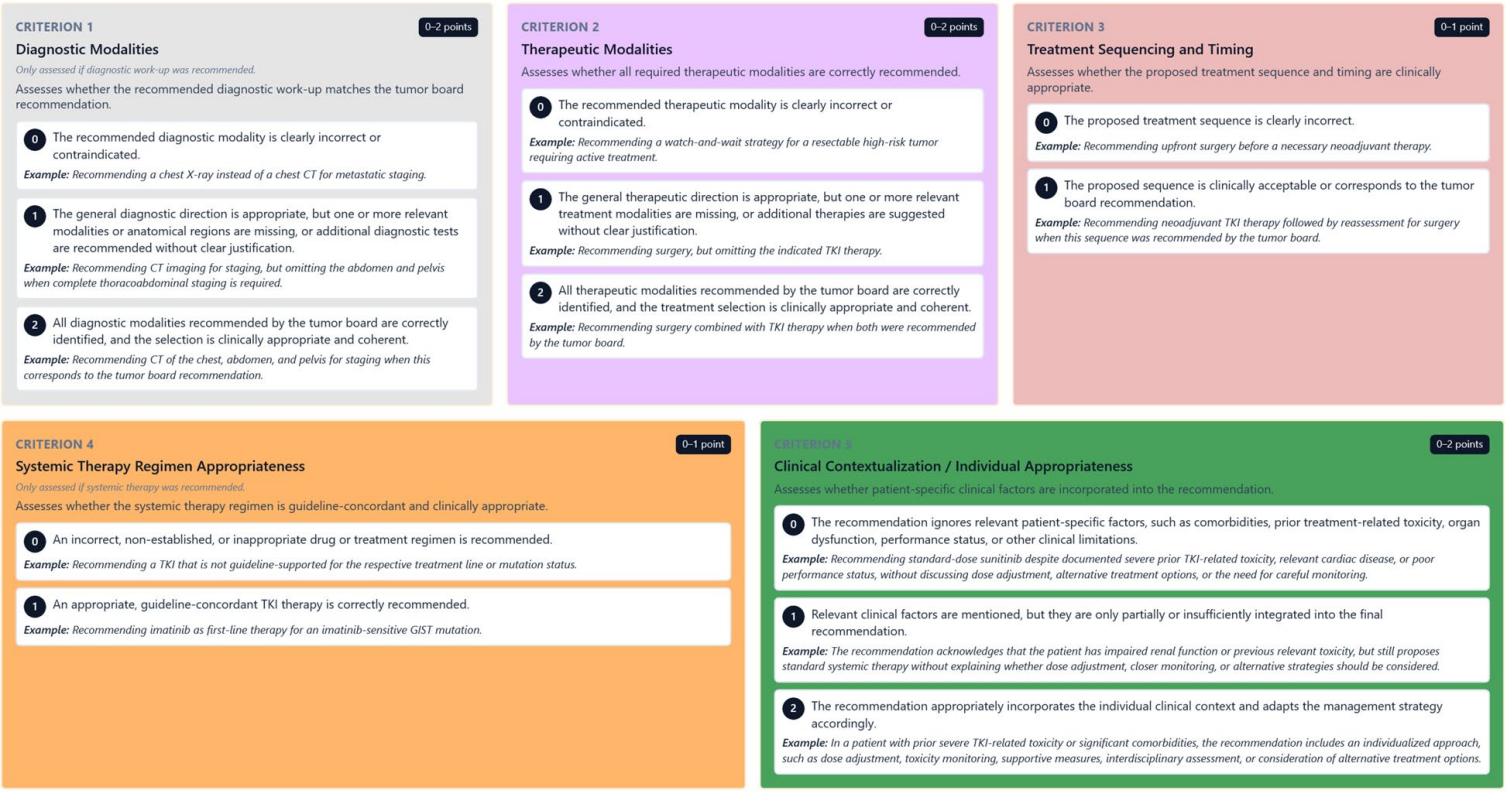
Evaluation criteria and scoring framework for expert assessment of LLM-generated GIST recommendations

To evaluate the clinical quality and relevance of the LLM-generated treatment recommendations, a predefined scoring framework consisting of five evaluation domains was applied. Each criterion was independently assessed in a blinded fashion by two expert reviewers with experience in GIST MTBs. Discrepancies between reviewers were resolved by consensus discussion. The evaluation framework included assessment of diagnostic recommendations, therapeutic modalities, treatment sequencing, systemic therapy regimen appropriateness, and clinical contextualization. A detailed overview of the scoring system and point allocation for each criterion is provided in Fig. 3.

### Statistical analysis

No sample size calculation was performed in advance due to the retrospective and descriptive design. For each model, criterion scores were normalized to a 0–1 scale by dividing the raw score by the criterion-specific maximum (C1/C2/C5: 0–2; C3/C4: 0–1). Criteria rated as not applicable were treated as missing and excluded from normalization. The total normalized score was calculated per case as the sum of applicable raw scores divided by the sum of applicable maxima. Descriptive statistics including mean, standard deviation, median, and interquartile range were reported for each criterion and for the total normalized score. Between-model comparisons were performed as paired analyses on the same cases. For each criterion, only cases with available scores in both models were included. Paired differences were tested using the Wilcoxon signed-rank test with two-sided hypothesis testing. Multiple testing across the five criterion-level comparisons was handled using the Holm procedure, while the total normalized score was analyzed separately. Within each model, differences across criteria were assessed using the Friedman test on complete cases, followed by pairwise Wilcoxon signed-rank tests with Holm adjustment. The proportion of perfect cases, defined as a total normalized score of 1.0, was compared between models using the exact McNemar test. Inter-rater agreement between the two expert reviewers was assessed before consensus resolution using weighted Cohen’s kappa, reported overall and per criterion. All tests were two-sided with a significance level of 0.05. Analyses were conducted in GraphPad Prism version 10.5.0.

## Results

### Study cohort and case characteristics

From 3,831 cases discussed in the institutional sarcoma tumor board registry, 300 cases were randomly selected using a computer-generated random number sequence. Among these, 105 cases were identified as GIST and assessed for eligibility. Five cases were utilized for prompt optimization and subsequently omitted from the primary analysis. An additional case was excluded owing to insufficient clinical documentation. Consequently, the definitive study population consisted of 99 GIST patients, all of which had been presented to the MTB for diagnostic validation and therapeutic strategy formulation. Within this cohort, the median age was 65 years (range: 21–91 years), with a sex distribution of 54 males (54.5%) and 45 females (45.5%). The most prevalent primary tumor anatomical locations included the stomach (*n =* 58), jejunum/ileum (*n =* 15), and metastatic/peritoneal GIST with unknown primary (*n =* 10). Comprehensive demographic data and tumor-related characteristics are delineated in Table 1.

**Table 1:** Demographic characteristics

Characteristics	Value
Number of Patients	99
Median Age, years (range)	65 (21–91)
Gender	
Male sex, n (%)	54 (54.5%)
Female sex, n (%)	45 (45.5%)
Primary Tumor Location	
Esophagus, n (%)	1 (1.0%)
Stomach, n (%)	58 (58.6%)
Duodenum, n (%)	7 (7.1%)
Jejunum/Ileum, n (%)	15 (15.2%)
Metastatic/Peritoneal GIST with unknown primary, n (%)	10 (10.1%)
Colorectal, n (%)	4 (4.0%)
Pancreas, n (%)	2 (2.0%)
Unknown*, n (%)	2 (2.0%)

^*^Case presented to the multidisciplinary tumor board without available pathological diagnosis at the time of discussion

### Between-model comparison of normalized scores

**Fig. 4:**
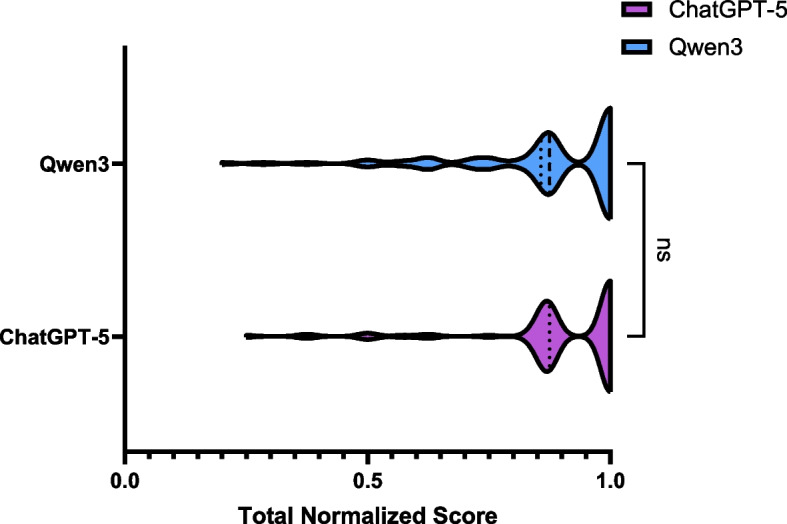
Comparison of total normalized scores between ChatGPT-5 and Qwen3. Violin plots show the distribution of case-level total normalized scores for each model. Dotted lines indicate the median and interquartile range. ns: not statistically significant (*p ≥* 0.05)

For the total normalized score, ChatGPT-5 achieved a mean of 0.901 ± 0.153 (median 1.000, IQR 0.875–1.000) and Qwen3 achieved a mean of 0.875 ± 0.171 (median 0.875, IQR 0.857–1.000) across 99 paired cases. The paired Wilcoxon test showed no significant difference (*p =* 0.451). Figure 4 illustrates the comparison of total normalized scores between ChatGPT-5 and Qwen3. At the criterion level, mean normalized scores were comparable between models, with ChatGPT-5 vs Qwen3 of 0.776 vs 0.730 for Diagnostics (C1; paired *n =* 87), 0.914 vs 0.889 for Therapeutics (C2; paired *n =* 99), 0.929 vs 0.929 for Treatment Sequence (C3; paired *n =* 99), 0.944 vs 0.986 for Systemic Therapy Regimen (C4; paired *n =* 73), and 0.965 vs 0.924 for Contextualization (C5; paired *n =* 99). After Holm correction across C1–C5, no criterion-level differences remained statistically significant (all adjusted *p*-values > 0.05). Descriptive statistics for the overall score and each individual evaluation criterion are summarized in Table 2.

**Table 2:** Descriptive statistics of model performance across evaluation criteria and overall score. (A) ChatGPT-5. (B) Qwen3

A						
**Criterion**	**Number of Evaluated Cases, n (%)**	Mean ± SD*	Median (IQR)**	**Score = 0 (%)**	**Score = 1 (%)**	**Score = 2 (%)**
Diagnostics (C1)	87 (87.9%)	0.776 ± 0.272	1.000 (0.500–1.000)	2 (2.3%)	35 (40.2%)	50 (57.5%)
Therapeutics (C2)	99 (100.0%)	0.914 ± 0.237	1.000 (1.000–1.000)	4 (4.0%)	9 (9.1%)	86 (86.9%)
Treatment Sequence (C3)	99 (100.0%)	0.929 ± 0.258	1.000 (1.000–1.000)	7 (7.1%)	92 (92.9%)	N/A***
Systemic Therapy Regimen (C4)	73 (73.7%)	0.945 ± 0.229	1.000 (1.000–1.000)	4 (5.5%)	69 (94.5%)	N/A***
Contextualization (C5)	99 (100.0%)	0.965 ± 0.129	1.000 (1.000–1.000)	0 (0.0%)	7 (7.1%)	92 (92.9%)
Total Normalized Score	99 (100.0%)	0.901 ± 0.153	1.000 (0.875–1.000)	N/A***	52 (52.5%)	N/A***

B						
**Criterion**	**Number of Evaluated Cases, n (%)**	Mean ± SD*	Median (IQR)**	**Score = 0 (%)**	**Score = 1 (%)**	**Score = 2 (%)**
Diagnostics (C1)	87 (87.9%)	0.730 ± 0.313	1.000 (0.500–1.000)	6 (6.9%)	35 (40.2%)	46 (52.9%)
Therapeutics (C2)	99 (100.0%)	0.889 ± 0.243	1.000 (1.000–1.000)	3 (3.0%)	16 (16.2%)	80 (80.8%)
Treatment Sequence (C3)	99 (100.0%)	0.929 ± 0.258	1.000 (1.000–1.000)	7 (7.1%)	92 (92.9%)	N/A***
Systemic Therapy Regimen (C4)	73 (73.7%)	0.986 ± 0.118	1.000 (1.000–1.000)	1 (1.4%)	71 (98.6%)	N/A***
Contextualization (C5)	99 (100.0%)	0.924 ± 0.207	1.000 (1.000–1.000)	2 (2.0%)	11 (11.1%)	86 (86.9%)
Total Normalized Score	99 (100.0%)	0.875 ± 0.171	0.875 (0.857–1.000)	N/A***	48 (48.5%)	N/A***

^*^Standard deviation

^**^Interquartile range

^***^Not applicable

### Within-model domain performance

**Fig. 5:**
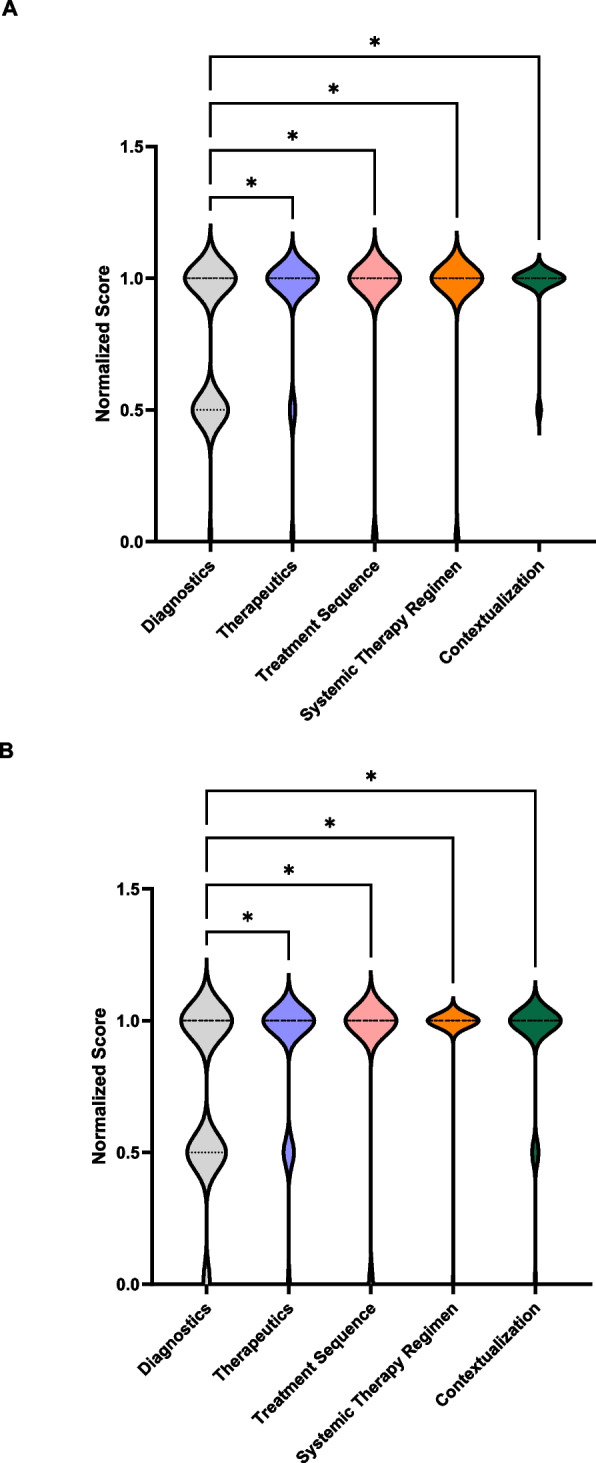
Within-model comparison of criterion-specific normalized scores for ChatGPT-5 (**A**) and Qwen3 (**B**). Dotted lines indicate the median and interquartile range. Asterisks (*) indicate statistically significant pairwise differences after adjustment (*p <* 0.05)

Within each model, normalized scores differed significantly across criteria, with a significant overall domain effect in both ChatGPT-5 and Qwen3 (Friedman test, both *p <* 0.05). Post hoc paired comparisons showed that Diagnostics (C1) was consistently lower than Therapeutics (C2), Treatment Sequence (C3), Systemic Therapy Regimen (C4), and Contextualization (C5) in both models (all adjusted *p*-values < 0.05). No further pairwise differences among C2–C5 were significant in either model (all adjusted *p*-values > 0.05). Figure 5A and B provide a criterion-wise comparison of normalized scores within ChatGPT-5 and Qwen3, respectively.

### Perfect-case analysis

**Fig. 6:**
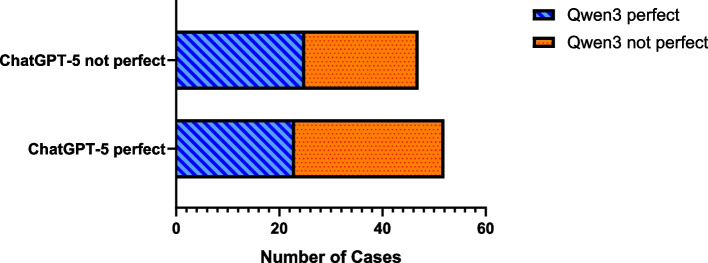
Distribution of perfect and non-perfect cases in the paired comparison between ChatGPT-5 and Qwen3

Perfect cases were defined as Total_norm = 1.0. In the 99 paired cases, ChatGPT-5 achieved a perfect score in 52/99 (52.5%) cases and Qwen3 in 48/99 (48.5%) cases. The paired contingency table showed 23 cases perfect in both models, 29 perfect only in ChatGPT-5, 25 perfect only in Qwen3, and 22 perfect in neither model. The exact McNemar test showed no significant difference in perfect-case rates between models (*p =* 0.684). Figure 6 shows the distribution of perfect and non-perfect cases across the two models.

### Inter‑rater agreement

Across 914 criterion-level ratings from both models, the overall inter-rater agreement between the two expert reviewers was almost perfect, with a Cohen’s kappa of 0.978 (percent agreement 98.8%). For Qwen3, agreement was almost perfect for Diagnostics (κ = 0.959) and Clinical Contextualization (κ = 0.861), with perfect agreement for Therapeutics (κ = 1.000) and Treatment Sequence and Timing (κ = 1.000) and Systemic Therapy Regimen Selection (κ = 1.000). For ChatGPT-5, agreement was almost perfect for Diagnostics (κ = 0.955), Therapeutics (κ = 0.914), and Clinical Contextualization (κ = 0.918), with perfect agreement for Treatment Sequence and Timing (κ = 1.000) and Systemic Therapy Regimen Selection (κ = 1.000).

## Discussion

The integration of artificial intelligence into clinical workflows is gaining momentum, with LLMs emerging as a promising tool for supporting medical decision-making. In the field of oncology, where individualized treatment strategies often depend on the collaborative insights of MTBs, LLMs may offer a scalable way to assist in complex case evaluations. Although MTBs have demonstrated clear benefits in optimizing care, their implementation is not always feasible due to constraints in time, expertise, and infrastructure [22]. These limitations have raised the question of whether LLMs could be used to complement existing MTB processes by providing preliminary, guideline-informed recommendations. In this study, we systematically evaluated the performance of two LLMs, one closed source (ChatGPT-5) and one open source (Qwen3), in generating treatment suggestions for GIST cases and assessed their consistency with decisions made by experienced MTB teams.

Our results demonstrated a high level of concordance between LLM-generated recommendations and expert MTB decisions in GIST management. Both models achieved high mean normalized scores, with no statistically significant differences observed between the closed-source and open-source models. These findings align with previous tumor board related studies indicating that LLMs demonstrate strong concordance with multidisciplinary tumor board recommendations in oncologic patient management [23–25].

In a recently published GIST tumor board study comparing ChatGPT with the open-source model DeepSeek-R1, the ChatGPT model achieved superior concordance with expert recommendations. In contrast, our findings showed comparable performance between the closed source ChatGPT-5 model and the open source Qwen3 model, suggesting that strong performance in MTB decision support may also be achievable with open-source architectures [26].

Our within-model analysis revealed a consistent domain-specific pattern. For both ChatGPT-5 and Qwen3, diagnostic recommendations achieved significantly lower scores than therapeutics, treatment sequencing, systemic therapy regimen selection, and clinical contextualization. This finding differs from our previous soft tissue sarcoma analysis (GISTs were excluded), in which clinical contextualization showed a particularly strong performance compared with other domains [15]. One possible explanation is the higher biological and therapeutic heterogeneity of soft tissue sarcomas, where individualized contextual factors may play a more prominent role in treatment planning. In contrast, GIST management is more strongly driven by defined variables such as mutation status, risk classification, resectability, and TKI sensitivity, which may allow LLMs to perform more consistently in therapeutic and contextual domains [27, 28]. At the same time, diagnostic decision-making in GIST often requires a more dynamic interpretation of the already available workup, including biopsy status, imaging adequacy, molecular testing, and local resectability assessment. Unlike therapeutic recommendations, which frequently follow relatively structured mutation- and stage-dependent pathways, diagnostic recommendations depend heavily on understanding what information is already present at the time of MTB referral. These context-dependent diagnostic considerations may be more difficult for LLMs to reconstruct from tumor board registration letters than guideline-based treatment algorithms [27, 29]. Additionally, we observed isolated cases of PET-CT over-recommendation, although this modality was not included in the guidelines on which the models were prompted to base their recommendations. This represents a form of confabulation, in which the model generates a clinically plausible but unsupported recommendation. Such errors may potentially be mitigated by retrieval-augmented generation systems that explicitly anchor model outputs to predefined guideline sources [30].

In the present study, perfect agreement with expert MTB recommendations was observed in 52.5% of cases for ChatGPT-5 and 48.5% for Qwen3, without a statistically significant difference between the models. Although these perfect-case rates appear lower than the overall concordance reported in previous GIST-related studies, they should be interpreted in the context of the stricter evaluation framework applied in our analysis [31]. A prior GIST tumor board study showed that overall treatment strategy concordance was higher than exact treatment concordance, highlighting the difference between general therapeutic agreement and complete recommendation matching [26]. Similarly, a recent study in urogenital oncology MTBs demonstrated that LLMs may approach expert-level performance in selected settings but still fall short of full equivalence with expert tumor board decisions [32]. Taken together, these findings suggest that while current LLMs are often capable of reproducing broad therapeutic strategies, exact replication of multidisciplinary oncologic decision-making remains substantially more challenging. Accordingly, the finding that nearly half of the evaluated cases showed at least one deviation from expert MTB recommendations underscores that current LLMs should be regarded as supportive tools rather than autonomous systems for multidisciplinary oncologic decision-making [33].

Comparison across published MTB studies should be interpreted with caution, as reported LLM performance varies substantially between cancer types, institutions, and study designs [32, 34]. This is consistent with recent evidence suggesting a complexity-dependent performance pattern, with higher concordance in standardized and common tumor entities and weaker performance in rare, complex, or multimodal clinical scenarios [35]. Several factors may contribute to this heterogeneity. First, studies often evaluate different model generations, which may differ considerably in reasoning capabilities and medical knowledge. Second, the nature and completeness of the clinical information provided to the model are rarely standardized and often insufficiently described. Since LLM outputs are highly dependent on the quality and structure of the input data, differences in referral letters, case summaries, and prompt design may substantially influence performance [36, 37]. Finally, MTB recommendations themselves do not always represent a direct translation of guideline recommendations. Although guidelines serve as an important foundation, expert MTBs frequently incorporate additional factors such as local expertise, patient preferences, comorbidities, resource availability, and individual clinical judgment [38–40]. Consequently, discordance between an LLM recommendation and an MTB decision should not necessarily be interpreted as model failure, just as concordance should not automatically imply superior clinical reasoning. Accordingly, comparisons of LLM performance across MTB studies should consider both the evaluation framework used and the specific domains in which agreement or disagreement with expert recommendations occurs.

Despite these heterogeneous and partly conflicting findings across published MTB studies, the performance observed in the present study supports a potential role for LLMs as assistive tools within oncology workflows. Rather than replacing expert decision-making, LLMs may be most useful in the preparatory phase of MTBs, where they could help structure fragmented patient histories, extract relevant clinical information, summarize diagnostic and therapeutic context, and highlight factors relevant to treatment planning [13, 41]. This may be particularly valuable in resource-limited settings or low-volume centers with restricted access to specialized GIST expertise. However, current evidence does not support autonomous use of LLMs in clinical practice [42, 43]. Their outputs remain dependent on the quality and completeness of the provided input, and ambiguous or incomplete case information may lead to information loss or clinically relevant errors [44]. Therefore, LLMs should currently be regarded as supportive technologies that may facilitate MTB preparation and organization, but require expert verification before any clinical implementation.

This study has several limitations. First, the analysis was performed at a single institution, and both MTB documentation style and clinical decision-making patterns may vary across centers. Therefore, the transferability of our findings to other GIST MTBs or broader oncology settings may be limited. Second, only cases with sufficient clinical information and documented MTB recommendations were included, introducing the possibility of selection bias. Third, MTB consensus decisions served as the reference standard for evaluation. While this reflects real-world clinical practice, MTB recommendations may also be influenced by institution-specific expertise, local resources, and individual patient factors that are not fully captured by formal guideline recommendations. Fourth, the input data consisted of German MTB registration letters, and it remains uncertain whether similar performance would be observed in other languages, healthcare systems, or documentation formats. Finally, we did not assess model variability across different parameter settings or repeated runs, which may influence output consistency and reproducibility.

In conclusion, both LLMs demonstrated a high degree of concordance with expert MTB recommendations in real-world GIST cases, with no significant differences between the closed-source and open-source model. Diagnostic recommendations represented the weakest domain, highlighting the challenge of reconstructing context-dependent diagnostic workflows from tumor board letters. These findings support the potential role of LLMs as assistive tools in GIST MTB workflows, particularly for structuring and synthesizing clinical information. However, human oversight remains essential. Further research, including multicenter investigations with more heterogeneous datasets, and evaluations of both domain-specific and next-generation LLMs, is needed to better define their role, generalizability, and suitability for integration into multidisciplinary oncology decision-making.

## Data Availability

The datasets generated and analyzed during the current study are not publicly available due to patient data protection and privacy but are available from the corresponding author on reasonable request.

## Abbreviations

GIST: Gastrointestinal Stromal Tumor
MTB: Multidisciplinary Tumor Board
AI: Artificial Intelligence
LLM: Large Language Model
GPT: Generative Pretrained Transformer
